# Photodynamic therapy induces autophagy-mediated cell death in human colorectal cancer cells via activation of the ROS/JNK signaling pathway

**DOI:** 10.1038/s41419-020-03136-y

**Published:** 2020-10-31

**Authors:** Changfeng Song, Wen Xu, Hongkun Wu, Xiaotong Wang, Qianyi Gong, Chang Liu, Jianwen Liu, Lin Zhou

**Affiliations:** 1 State Key Laboratory of Bioreactor Engineering and Shanghai Key Laboratory of New Drug Design, School of Pharmacy, East China University of Science and Technology, Shanghai, 200237 P.R. China; 2 Department of Laboratory Medicine, Changzheng Hospital, Naval Medical University, Shanghai, 200003 P.R. China

**Keywords:** Cancer therapy, Autophagy

## Abstract

Evidence has shown that m-THPC and verteporfin (VP) are promising sensitizers in photodynamic therapy (PDT). In addition, autophagy can act as a tumor suppressor or a tumor promoter depending on the photosensitizer (PS) and the cancer cell type. However, the role of autophagy in m-THPC- and VP-mediated PDT in in vitro and in vivo models of human colorectal cancer (CRC) has not been reported. In this study, m-THPC-PDT or VP-PDT exhibited significant phototoxicity, inhibited proliferation, and induced the generation of large amounts of reactive oxygen species (ROS) in CRC cells. From immunoblotting, fluorescence image analysis, and transmission electron microscopy, we found extensive autophagic activation induced by ROS in cells. In addition, m-THPC-PDT or VP-PDT treatment significantly induced apoptosis in CRC cells. Interestingly, the inhibition of m-THPC-PDT-induced autophagy by knockdown of ATG5 or ATG7 substantially inhibited the apoptosis of CRC cells. Moreover, m-THPC-PDT treatment inhibited tumorigenesis of subcutaneous HCT116 xenografts. Meanwhile, antioxidant treatment markedly inhibited autophagy and apoptosis induced by PDT in CRC cells by inactivating JNK signaling. In conclusion, inhibition of autophagy can remarkably alleviate PDT-mediated anticancer efficiency in CRC cells via inactivation of the ROS/JNK signaling pathway. Our study provides evidence for the therapeutic application of m-THPC and VP in CRC.

## Introduction

Colorectal cancer (CRC) is the third leading cause of cancer death globally, with a high incidence and mortality rate^1^. CRC is stratified into two subgroups: early stage (stage I and II) and advanced-stage (stage III and IV)^2^. The 5-year survival rate for patients diagnosed with early stage CRC is approximately 90%, whereas the survival rate for patients diagnosed with advanced-stage CRC is as low as 13.1%^3^. Surgical resection is the main treatment method for patients with early stage CRC, while chemotherapy is regarded as the primary treatment option for patients with advanced-stage CRC^2,4^. Despite the improvement in the treatment of CRC, the mortality rate of CRC is still high. Thus, there is an urgent need to develop alternative treatments for CRC.

Photodynamic therapy (PDT) is a minimally invasive, effective cancer treatment modality that has emerged as an alternative or additional approach to chemotherapy and surgery^5^. PDT has been clinically available and approved to treat some types of cancers, such as head and neck cancer, non-small cell lung cancer, prostate cancer, and colon cancer^6–9^. PDT involves three primary components, namely a nontoxic photosensitizer (PS), a light source, and oxygen^10^. During PDT, PSs absorb visible light and convert energy to surrounding molecular oxygen and generate a range of highly reactive oxygen species (ROS), such as singlet oxygen, superoxide anions, and hydroxyl radicals^11,12^. High levels of ROS can rapidly cause significant toxicity, which eventually leads to cell death via apoptosis, autophagy, and/or necrosis^13,14^. PSs function as catalysts during the process of PDT^15^. Meta-tetrahydroxyphenylchlorin (m-THPC) and verteporfin (VP) are second-generation photosensitizers that exhibit considerable photocytotoxicity to various tumor cells^16,17^. Emerging studies have found that m-THPC-PDT and VP-PDT could be promising therapeutic candidates for the treatment of human cancers^18,19^.

The role of a PS in the PDT process is similar to that of chemical catalysts^10^. It can be excited by specific wavelengths of light and absorb the energy of photons, converting them from a stable ground state to a high-energy excited singlet state. Singlet oxygen generates free radicals in the process of returning to the ground state, and free radicals react with molecular oxygen to generate ROS^20^. A variety of PSs exist in nature, but the PSs used for tumor treatment are demanding: they need to have the characteristics of high singlet oxygen yield, non-toxicity, rapid elimination from the body through metabolism, and easy accumulation in tumor tissues^21,22^. The PSs used in PDT can be divided into porphyrin derivative PS, chlorophyll-derivative PS, and synthetic compound PS^23^. According to the time of occurrence, it can be divided into first-generation, second-generation, and third-generation PS^24^. Choosing the right PS to treat a specific disease is particularly critical. The properties of PS, such as charge and polarity, are critical to their cellular localization, distribution in the body, and therapeutic efficacy. Many PSs selectively accumulate in specific organelles, such as late endosomes, lysosomes, mitochondria, or the endoplasmic reticulum^25^. In this case, light causes photo damage to specific organelles. Therefore, determining the location of the PS in the cell will provide a better understanding of the site of action of phototoxicity^26^.

Autophagy is a successive process of degrading and renewing cytoplasmic components^27^. In addition, it is critical for maintaining homeostasis and cell growth^28^. Evidence has shown that autophagy participates in tumor progression as well as a response to anticancer therapies^29^. It has also been shown that photodamage can lead to autophagy induction^29,30^. However, autophagy might play dual roles in tumor suppression and promotion depending on the photosensitizers, cell types, and light dose used^31^. Xiong et al. reported that inhibition of autophagy increased photocytotoxicity of Photosan-II in CRC cells^32,33^. Meanwhile, Xue et al. indicated that knockdown of ATG7 enhanced the resistance of breast cancer cells to PDT^33^. In this study, we aimed to investigate whether m-THPC-PDT or VP-PDT treatment has an anticancer effect in CRC cells and explore the roles of autophagy in PDT treatment in CRC.

## Materials and methods

### Cell culture

Human colon cancer cell lines HCT116 and SW480 were purchased from the American Type Culture Collection (ATCC, USA). HCT116 cells were cultured in RPMI 1640 medium (Thermo Fisher Scientific, Waltham, MA, USA) supplemented with 10% fetal bovine serum (Thermo Fisher Scientific) and 1% penicillin/streptomycin (HyClone, Logan, UT, USA). SW480 cells were maintained in Dulbecco’s modified Eagle’s medium (Thermo Fisher Scientific) supplemented with 10% fetal bovine serum (HyClone) and 1% penicillin/streptomycin. Cells were incubated in a humidified atmosphere of 5% CO_2_ at 37 °C. For starvation experiments, cells were washed three times with phosphate-buffered saline (PBS), and once with serum-free media, and then incubated with modified Earle’s Balanced Salt Solution medium (Sigma-Aldrich, St. Louis, MO, USA) containing 1.0 g/L glucose at 37 °C.

### M-THPC-PDT and VP-PDT

The chemicals m-THPC and VP were purchased from MedChemExpress (Monmouth Junction, NJ, USA). They were dissolved in DMSO as a 10 mmol/L stock solution and stored at −80 °C, protected from light. Working solutions of m-THPC were prepared fresh at various concentrations (0, 0.375, 0.75, 1.5, 3.0, 6.0, and 12.0 μmol/L) and working solutions of VP were prepared fresh at various concentrations (0, 0.375, 0.75, 1.5, 3.0, and 6.0 μmol/L), which were activated by a halogen lamp in cell experiments, a laser power density of 10 mW/cm^2^ and laser energy densities of 3 J/cm^2^.

### Cell viability assay

An MTT Cell Proliferation and Cytotoxicity Assay Kit (Beyotime Biotechnology, Shanghai, China) was used to determine cell viability. HCT116 and SW480 cells (6 × 10^3^ cells per well) were seeded into 96-well plates and incubated overnight at 37 °C. After that, HCT116 and SW480 cells were treated with various concentrations of m-THPC (from 0 to 12.0 μmol/L) or VP (from 0 to 6.0 μmol/L) for 8 h. Next, cells were irradiated with a light dose of 3 J/cm^2^ at a light power density of 10 mW/cm^2^, and then incubated for 24 h without irradiation. Subsequently, cells were incubated with 20 μL of 5 mg/mL MTT for another 4 h. Subsequently, formazan crystals were dissolved using 150 μL of formazan dissolution reagent. Finally, the optical density value of each well at 570 nm was measured using a microplate reader (BioTek, Winooski, VT, USA).

### Measurement of intracellular ROS level

HCT116 and SW480 cells were incubated in the corresponding medium with 10 µmol/L 2′,7′-Dichlorodihydrofluorescein diacetate (H_2_DCFDA) (MedChemExpress) for 30 min at 37 °C in the dark, and then cells were washed twice with PBS and digested with trypsin. Finally, the fluorescent intensity of intracellular ROS was detected by flow cytometry (Beckman Coulter CyAn^™^ ADP).

### Western blot

Aliquots of 30 μg of protein were subjected to 12.5% sodium dodecyl sulfate polyacrylamide gel electrophoresis and then transferred onto a polyvinylidene fluoride membrane (Millipore, Billerica, MA, USA). After that, the membrane was blocked with 5% non-fat milk in tris-buffered saline containing Tween 20 for 1 h at room temperature. Later on, the membrane was incubated overnight at 4 °C with the following primary antibodies against: MAP1LC3B obtained from CST (Cell Signaling Technologies, Danvers, MA, USA, 1:1000, #4108S), SQSTM1/p62 (1:1000, #5114S, CST), ATG5 (1:1000, #12994S, CST), ATG7 (1:1000, #2631S, CST), p-JNK (Thr183/Tyr185, 1:1000, #4668S, CST), JNK (1:1000, #9258S, CST), p-p70S6K (Thr389, 1:1000, #9234S, CST), p70S6K (1:1000, #2708S, CST), p-mTOR (Ser2481, 1:1000, #2974S, CST), p-mTOR (Ser2448, 1:1000, #2971S, CST), mTOR (1:1000, #2983S, CST), and β-actin (1:1000, #4970L, CST). Subsequently, the membrane was incubated with the corresponding secondary antibody labeled with horseradish peroxidase (#7074, CST) at room temperature for 1 h. Finally, the protein bands were visualized using an enhanced chemiluminescence substrate kit (Thermo Fisher Scientific), and analyzed using Image J software (National Institutes of Health, Bethesda, MA, USA).

### Fluorescence microscopy

The Stub-RFP-Sens-GFP-MAP1LC3B lentivirus was obtained from GeneChem (GVAP01689345). HCT116 or SW480 cells were infected with lentiviral constructs to construct stably expressing Stub-RFP-Sens-GFP-MAP1LC3B cell lines. When analyzed for GFP-MAP1LC3B autophagic aggregation spots, cells were cultured on cell climbing slices, washed with PBS, fixed with 4% paraformaldehyde for 10 min, stained with DAPI (Sigma-Aldrich) for 5 min at room temperature, and protected from light. Finally, the cells were observed with a Leica SP8 confocal microscope (Leica Microsystems Inc., Germany) using a 63× oil immersion lens.

### Transmission electron microscopy

HCT116 and SW480 cells were harvested and fixed with 2.5% glutaraldehyde at 4 °C overnight. Cells were then fixed in 1% osmium tetroxide (OsO_4_) in 0.1 mol/L PBS (pH 7.4) for 2 h at room temperature. After washing three times with PBS, the cells were dehydrated in a graded series of ethanol solutions for 15 min. Subsequently, cells were cut into ultra-thin sections (100 nm) and stained with uranyl acetate and lead citrate. Finally, images were observed under a transmission electron microscope (H-7650, Hitachi, Japan).

### Cyto-ID green analysis of autophagy

The Cyto-ID Autophagy Detection Kit (Enzo Life Sciences, NY, USA) was used to detect cell autophagy according to the manufacturer’s protocol. Autophagy analysis was performed using flow cytometry, as described previously^34^.

### Cell apoptosis

HCT116 and SW480 cells were washed twice with PBS and then suspended in binding buffer (Thermo Fisher Scientific), followed by Annexin V-FITC and PI (Thermo Fisher Scientific) staining for 15 min. Finally, the percentage of apoptotic cells was determined using flow cytometry.

### Small interfering RNA

SiATG5 and siATG7 were purchased from Ribobio (Guangdong, China). The target sequences were as follows: ATG5: 5ʹ-GCUCUUCCUUGGAACAUCA-3ʹ; ATG7: 5ʹ-CAACAUCCCUGGUUACAAG-3ʹ. HCT116 and SW480 cells were transiently transfected with 50 nM siATG5 or siATG7 using riboFECT^™^ CP Reagent (Ribobio, Guangdong, China) according to the manufacturer’s instructions.

### Lentivirus production and transduction

Lentiviral shRNA constructs targeting ATG7 were purchased from Hanbio (Shanghai, China). The target sequences were as follows: top strand, 5ʹ-GATCCGCAACATCCCTGGTTACAAGTTCAAGAGACTTGTAACCAGGGATGTTGTTTTTTG-3ʹ; bottom strand: 5ʹ-AATTCAAAAAACAACATCCCTGGTTACAAGTCTCTTGAACTTGTAACCAGGGATGTTGCG-3ʹ. 293T cells were transfected with the shRNA-expression vector and packaging plasmids using Lipofectamine2000 (Thermo Fisher Scientific). After that, virus-containing supernatants were collected at 48 h, and then passed through a 0.45-micron filter to obtain viral particles. Later, the viral supernatant fraction was added to CRC cells in the presence of Polybrene (Santa Cruz Biotechnology, Santa Cruz, CA, USA). Subsequently, 72 h after transduction, the infected cells were selected with puromycin (2 µg/mL, Abcam).

### Animal study

Five-week-old male BALB/c mice were purchased from the Shanghai Experimental Animal Center of Chinese Academic of Sciences (Shanghai, China). The experimental protocol was initially approved by the Animal Care and Use Committee of the Second Military Medical University, and the animal experiments were conducted under the National Institute Guide for the Care and Use of Laboratory Animals. Totally, 1 × 10^6^ HCT116-control or HCT116-shATG7 cells suspended in 100 μL PBS were injected subcutaneously into the right back of the mice. The tumor size of each animal was measured using Vernier calipers every two days, and the total volume was calculated as *V*: volume = (length × width^2^)/2. When the tumors reached an average volume of 250 mm^3^, the mice were randomly divided into a non-PDT/control knockdown group, a PDT/control knockdown group, a non-PDT/ATG7 knockdown group, and a PDT/ATG7 knockdown group (*n* = 12). M-THPC was injected into the caudal vein at a dose of 0.1 mg/kg. Forty-eight hours later, mice were irradiated with a 20 J/cm^2^ light dose at a wavelength of 650 nm for 100 s with a laser power density of 200 mW/cm^2^. After 96 h of m-THPC-PDT treatment, 6 mice per group were sacrificed with excess CO_2_, and the tumor tissues were taken, fixed, embedded in paraffin for immunohistochemical experiments. After 14 days, the mice were sacrificed, and the entire tumor was weighed. M-THPC was dissolved in a mixture of polyethylene glycol-400: ethanol: water (3:2:5 by volume) as described previously^35^. The 650 nm laser source equipment was from Changchun New Industries Optoelectronics Technology (MRL-III-650, Changchun, China).

### Immunohistochemical (IHC) analysis

The tumor tissues were fixed in 4% paraformaldehyde and then embedded in paraffin. The samples were sliced into 5-μm-thick sections. The sections were then incubated with primary antibodies (ATG7, MAP1LC3B, SQSTM1/p62, CST) for 16 h at 4 °C. The sections were then incubated with horseradish peroxidase-conjugated goat anti-rabbit secondary antibody (Abcam) for 1 h, and visualized using diaminobenzidine (DAB) solution (Thermo Fisher Scientific). Finally, the images were observed with a LEICA DMi8 inverted fluorescence microscope (Leica Microsystems Inc., Germany).

### Statistical analysis

All experiments were repeated three times. Data are expressed as the mean ± standard deviation. All statistical analyses were performed using GraphPad Prism software (version 7.0, La Jolla, CA, USA). One-way analysis of variance and Tukey’s tests were performed for multiple group comparisons. *P < 0.05 was considered statistically significant.

## Results

### M-THPC-PDT and VP-PDT presented strong phototoxicity on CRC cells

**Fig. 1:**
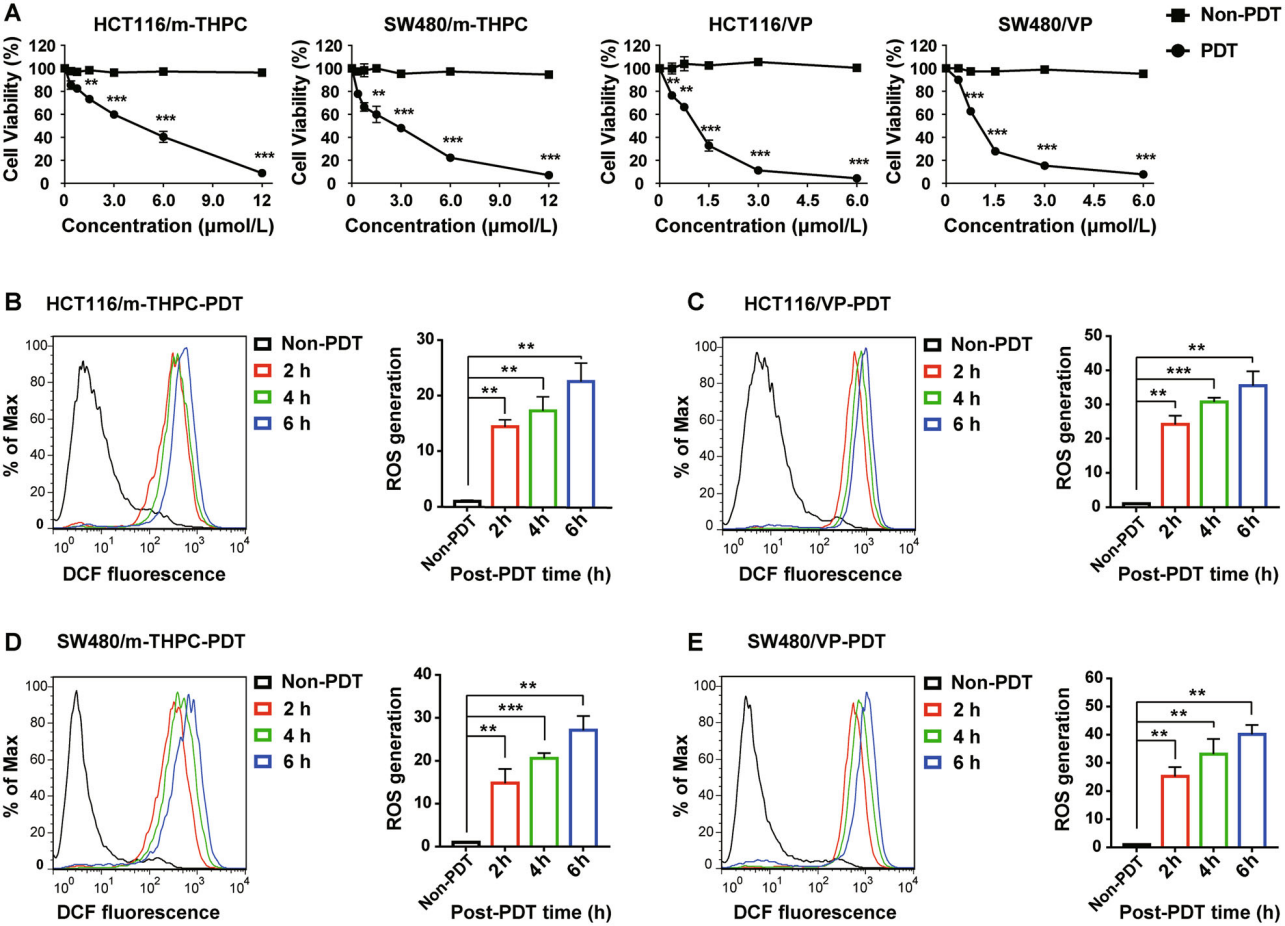
M-THPC-PDT and VP-PDT presented a strong phototoxic effect on CRC cells. **A** HCT116 and SW480 cells were treated with various concentrations of m-THPC (0–12.0 μmol/L) or VP (0–6.0 μmol/L) for 8 h, respectively, and then irradiated with a light dose of 3 J/cm^2^, followed by incubation without irradiation for 24 h. Cell viability was detected by an MTT assay. **B**, **C** HCT116 and **D**, **E** SW480 cells were treated with m-THPC (final concentration, 0.7 μmol/L) or VP (final concentration, 0.35 μmol/L), respectively, for 8 h, and then irradiated with a light dose of 3 J/cm^2^, followed by incubation without irradiation for another 2, 4, and 6 h. ROS production in HCT116 and SW480 cells was detected by flow cytometry. All data are presented as the mean ± SD, *n* = 3. ^**^P < 0.01, ^***^P < 0.001.

To investigate the effects of m-THPC-PDT and VP-PDT on the viability of HCT116 and SW480 cells, MTT assay was performed. As indicated in Fig. 1A, m-THPC-PDT or VP-PDT treatment inhibited the proliferation of HCT116 and SW480 cells in a dose-dependent manner. In previous studies, we found that 0.7 μmol/L m-THPC and 0.35 μmol/L VP could maximize the activation of autophagy in HCT116 and SW480 cells at a light dose of 3 J/cm^2^. Therefore, 0.7 μmol/L m-THPC or 0.35 μmol/L VP was utilized in the following experiments. In addition, ROS production was measured by flow cytometry. As shown in Fig. 1B–E, ROS production increased in HCT116 and SW480 cells in a time-dependent manner after m-THPC-PDT or VP-PDT treatment. These data indicated that m-THPC-PDT or VP-PDT had a strong phototoxic effect on CRC cells.

### M-THPC-PDT-induced autophagy in CRC cells

**Fig. 2:**
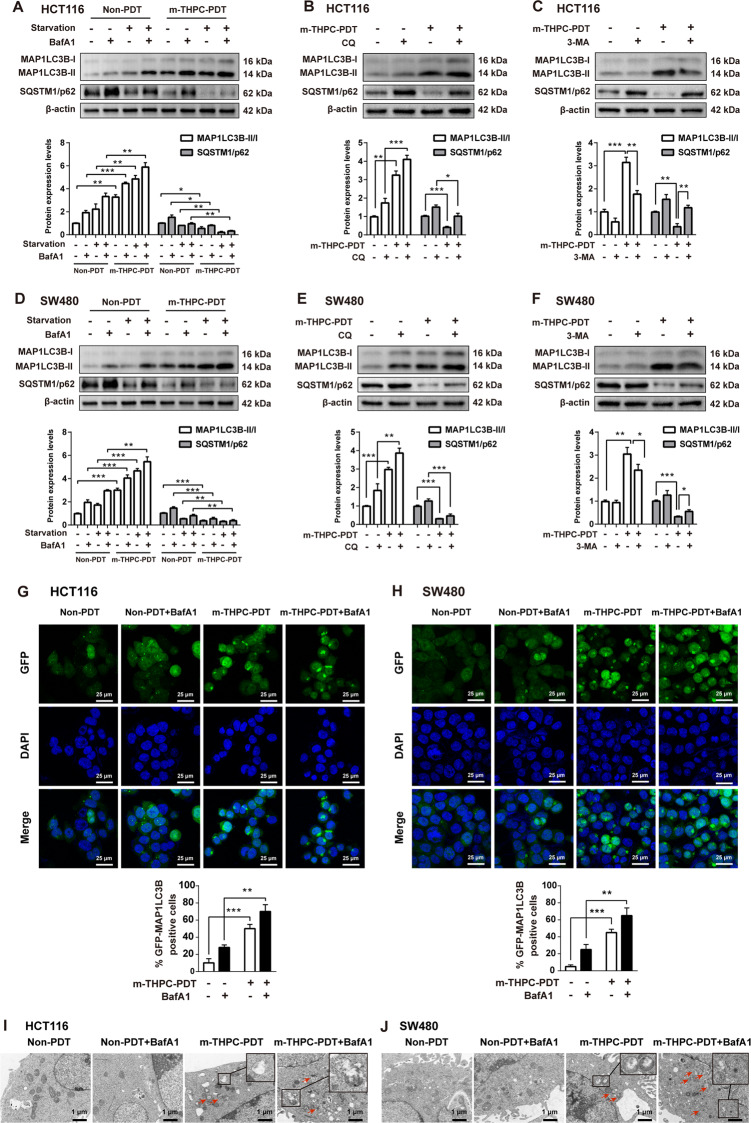
M-THPC-PDT induced autophagy in CRC cells. **A**–**C** HCT116 and **D**–**F** SW480 cells were treated with 0.7 μmol/L m-THPC for 8 h, and then irradiated with a light dose of 3 J/cm^2^, followed by incubation without irradiation for 8 h. Subsequently, cells were treated with 10 nmol/L BafA1 for 4 h or treated with 50 μmol/L CQ for 4 h after the PDT treatment. Cells were treated with 5 mmol/L 3-MA 12 h prior to the PDT treatment. For starvation experiments, cells were incubated for 4 h in serum-free media after the PDT treatment, followed by incubation with 10 nmol/L of BafA1 for 4 h. Western blot analysis of MAP1LC3B-I, MAP1LC3B-II, and SQSTM1/p62 levels in HCT116 and SW480 cells. **G**, **H** GFP-MAP1LC3B puncta were observed by immunofluorescence using a laser scanning confocal microscope. **I**, **J** The images of the autophagic vacuoles (the red arrow) observed under TEM. All data are presented as the mean ± SD, *n* = 3. ^**^P < 0.01, ^***^P < 0.001.

To investigate whether autophagy is triggered by m-THPC-PDT, the conversion of MAP1LC3B-I to MAP1LC3B-II and the level of SQSTM1/p62 were measured. As shown in Fig. 2A–F, m-THPC-PDT markedly increased the accumulation of MAP1LC3B-II and decreased the accumulation of SQSTM1/p62 in HCT116 and SW480 cells, indicating that m-THPC-PDT induced autophagy in CRC cells. In addition, the expression of MAP1LC3B-II was increased, but the expression of SQSTM1/p62 was decreased in HCT116 and SW480 cells grown in serum-starved medium, indicating that starvation induced cell autophagy (Fig. 2A, D). As expected, starvation-induced autophagy was further enhanced in CRC cells after m-THPC-PDT (Fig. 2A, D).

Next, three autophagy inhibitors, bafilomycin A1 (BafA1), chloroquine (CQ), and 3-methyladenine (3-MA) were used to confirm whether m-THPC-PDT induced cell autophagy. Treatment with BafA1 and CQ, two inhibitors of the autophagy-lysosomal pathway, led to a strong accumulation of MAP1LC3B-II and SQSTM1/p62 in HCT116 and SW480 cells (Fig. 2B, E)^36^. An inhibitor of class III PI3K, 3-MA suppressed the conversion of MAP1LC3B-I to MAP1LC3B-II and elevated the accumulation of SQSTM1/p62 in HCT116 and SW480 cells (Fig. 2C, F). As shown in Fig. 2A, B, D, E, the inductive role of m-THPC-PDT on autophagy in HCT116 and SW480 cells was reversed by treatment with BafA1 or CQ, as shown by the increased levels of MAP1LC3B-II and SQSTM1/p62. Consistently, 3-MA treatment significantly reversed m-THPC-PDT-induced autophagy, as shown by a decreased accumulation of MAP1LC3B-II and an increased expression of SQSTM1/p62 in CRC cells (Fig. 2C, F).

Immunofluorescence assays revealed that m-THPC-PDT induced an increase in the number of GFP-MAP1LC3B puncta in CRC cells, which was further enhanced in the presence of BafA1 (Fig. 2G, H). In addition, the typical structures of autophagic vacuoles were detected using TEM. Normal morphology and no autophagosomes were observed in non-PDT treated CRC cells, whereas double membrane-bound vacuoles without ribosomes were observed in m-THPC-PDT-treated CRC cells (Fig. 2I, J). These results indicated that m-THPC-PDT could induce autophagy in CRC cells.

### VP-PDT induced autophagy in CRC cells

**Fig. 3:**
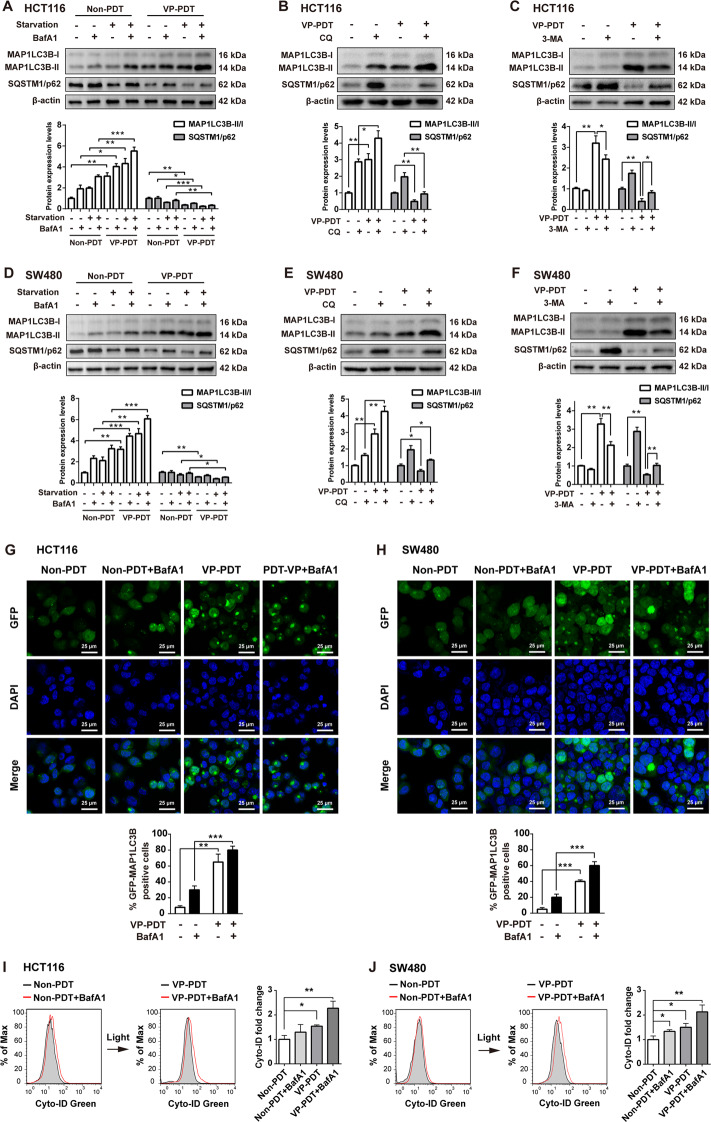
VP-PDT induced autophagy in CRC cells. **A**–**C** HCT116 and **D**–**F** SW480 cells were treated with 0.35 μmol/L VP for 8 h, and then irradiated with a light dose of 3 J/cm^2^, followed by incubation without irradiation for 8 h. Subsequently, cells were treated with 10 nmol/L BafA1 for 4 h or treated with 50 μmol/L CQ for 4 h, after the PDT treatment. Cells were treated with 5 mmol/L 3-MA 12 h prior to PDT treatment. For starvation experiments, cells were incubated for 4 h in serum-free media after PDT treatment, followed by incubation with 10 nmol/L of BafA1 for 4 h. Western blot analysis of MAP1LC3B-I, MAP1LC3B-II, and SQSTM1/p62 levels in HCT116 and SW480 cells. **G**, **H** GFP-MAP1LC3B puncta were observed by immunofluorescence using a laser scanning confocal microscope. **I**, **J** Cell autophagy was examined by a Cyto-ID Autophagy Detection Kit. All data are presented as the mean ± SD, *n* = 3. ^*^P < 0.05, ^**^P < 0.01, ^***^P < 0.001.

To investigate whether autophagy is triggered by VP-PDT, the conversion of MAP1LC3B-I to MAP1LC3B-II and the level of SQSTM1/p62 were measured. As shown in Fig. 3A–F, VP-PDT significantly increased the accumulation of MAP1LC3B-II and decreased accumulation of SQSTM1/p62 in CRC cells. In addition, starvation-induced autophagy was further enhanced in CRC cells after VP-PDT (Fig. 3A, D).

Next, three autophagy inhibitors, BafA1, CQ, and 3-MA, were used to confirm whether VP-PDT-induced autophagy in CRC cells. As indicated in Fig. 3A, B, D, E, the inductive role of VP-PDT on autophagy in CRC cells was reversed by treatment with BafA1 or CQ, as shown by the increased levels of MAP1LC3B-II and SQSTM1/p62. Consistently, 3-MA treatment remarkably attenuated VP-PDT-induced autophagy, as evidenced by a decreased autophagic MAP1LC3B-II/MAP1LC3B-I ratio and increased expression of SQSTM1/p62 in CRC cells (Fig. 3C, F).

In addition, cell autophagy was further examined by an immunofluorescence assay and the Cyto-ID Autophagy Detection Kit. As indicated in Fig. 3G–J, VP-PDT treatment caused a significant increase in autophagic flux in CRC cells. As expected, VP-PDT-induced autophagic flux was further enhanced in the presence of BafA1 (Fig. 3G–J). These data indicated that VP-PDT could induce autophagy in CRC cells.

### NAC treatment decreased the autophagy and apoptosis induced by m-THPC-PDT in CRC cells

**Fig. 4:**
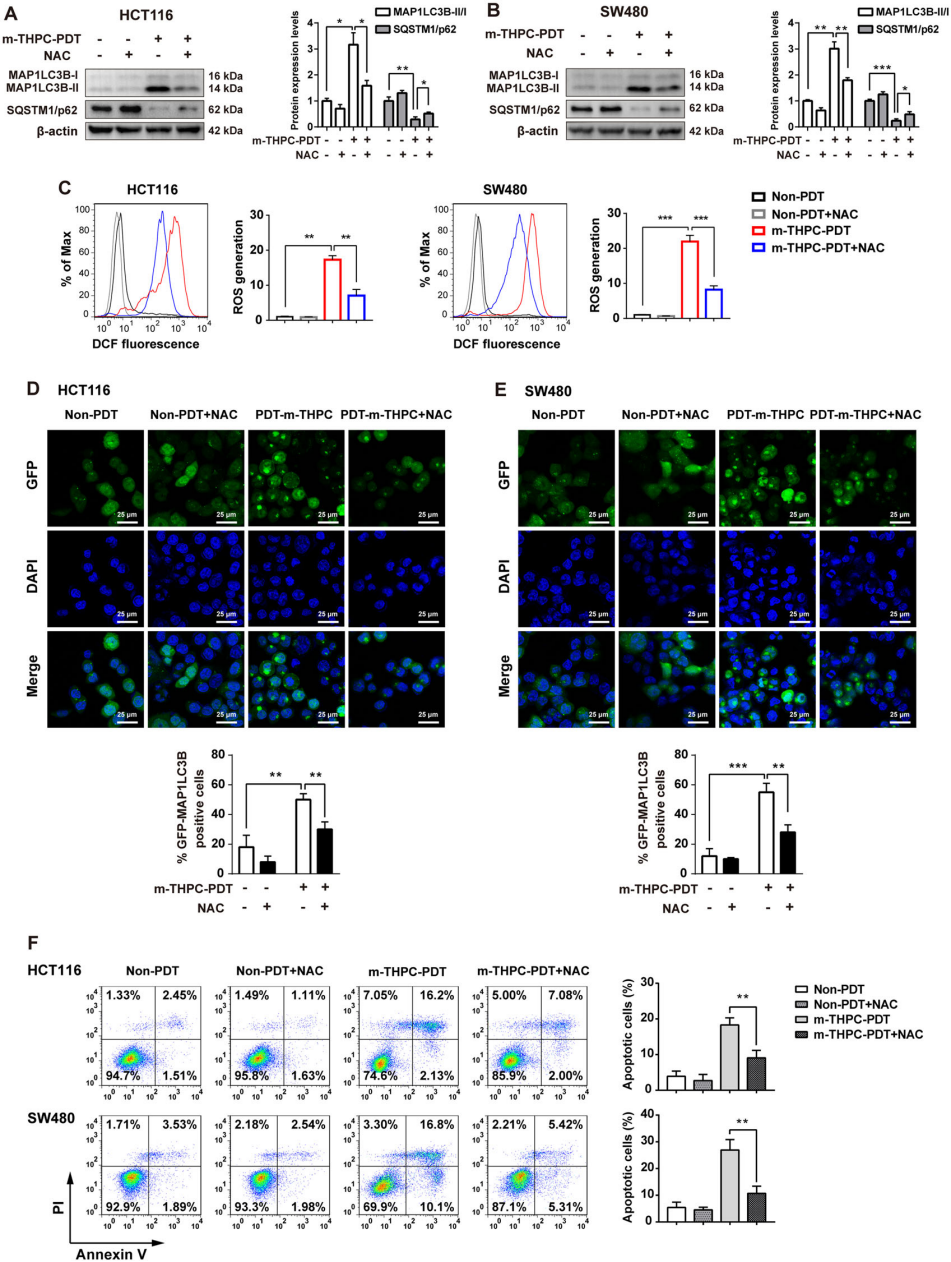
NAC treatment decreased the autophagy and apoptosis induced by m-THPC-PDT in CRC cells. HCT116 and SW480 cells were treated with 0.7 μmol/L m-THPC in the presence or absence of 5 mmol/L NAC. NAC and m-THPC were added to the medium 2 and 8 h before irradiation, respectively, and then irradiated with a light dose of 3 J/cm^2^. Followed by incubation without irradiation for 8 h, **A**, **B** Western blot analysis of MAP1LC3B-I, MAP1LC3B-II, and SQSTM1/p62 levels in HCT116 and SW480 cells. **C** The level of ROS production in HCT116 and SW480 cells was analyzed by flow cytometry to detect DCF fluorescence intensity. **D**, **E** GFP-MAP1LC3B puncta were observed by immunofluorescence using a laser scanning confocal microscope. **F** After 24 h of radiation, flow cytometry was performed to measure cell apoptosis with Annexin V-FITC and PI staining. All data are presented as the mean ± SD, *n* = 3. ^**^P < 0.01, ^***^P < 0.001.

In view of the fact that m-THPC-PDT treatment could induce autophagy in CRC cells and that m-THPC-PDT treatment markedly induced ROS generation in cells, we sought to explore the interaction between autophagy and ROS in m-THPC-PDT-treated CRC cells. Evidence has shown that N-acetyl cysteine (NAC), a ROS scavenger, was employed as a thiol antioxidant^37^. To assess the role of ROS in modulating autophagy in m-THPC-PDT-treated CRC cells, CRC cells were pre-treated with m-THPC and NAC for 8 h before exposure to PDT. As shown in Fig. 4A–C, treatment with NAC resulted in reduced ROS levels, decreased expression of MAP1LC3B-II, and accumulation of SQSTM1/p62 in m-THPC-PDT-treated CRC cells. In addition, the immunofluorescence assay indicated that NAC markedly abolished the formation of GFP-MAP1LC3B puncta in m-THPC-PDT-treated CRC cells (Fig. 4D, E). Moreover, m-THPC treatment significantly induced apoptosis in CRC cells, which was notably reversed in the presence of NAC (Fig. 4F). These data indicated that antioxidant treatment could suppress autophagy and apoptosis induced by m-THPC-PDT in CRC cells.

### Knockdown of ATG5 or ATG7 relieved autophagy and apoptosis induced by m-THPC-PDT in CRC cells

**Fig. 5:**
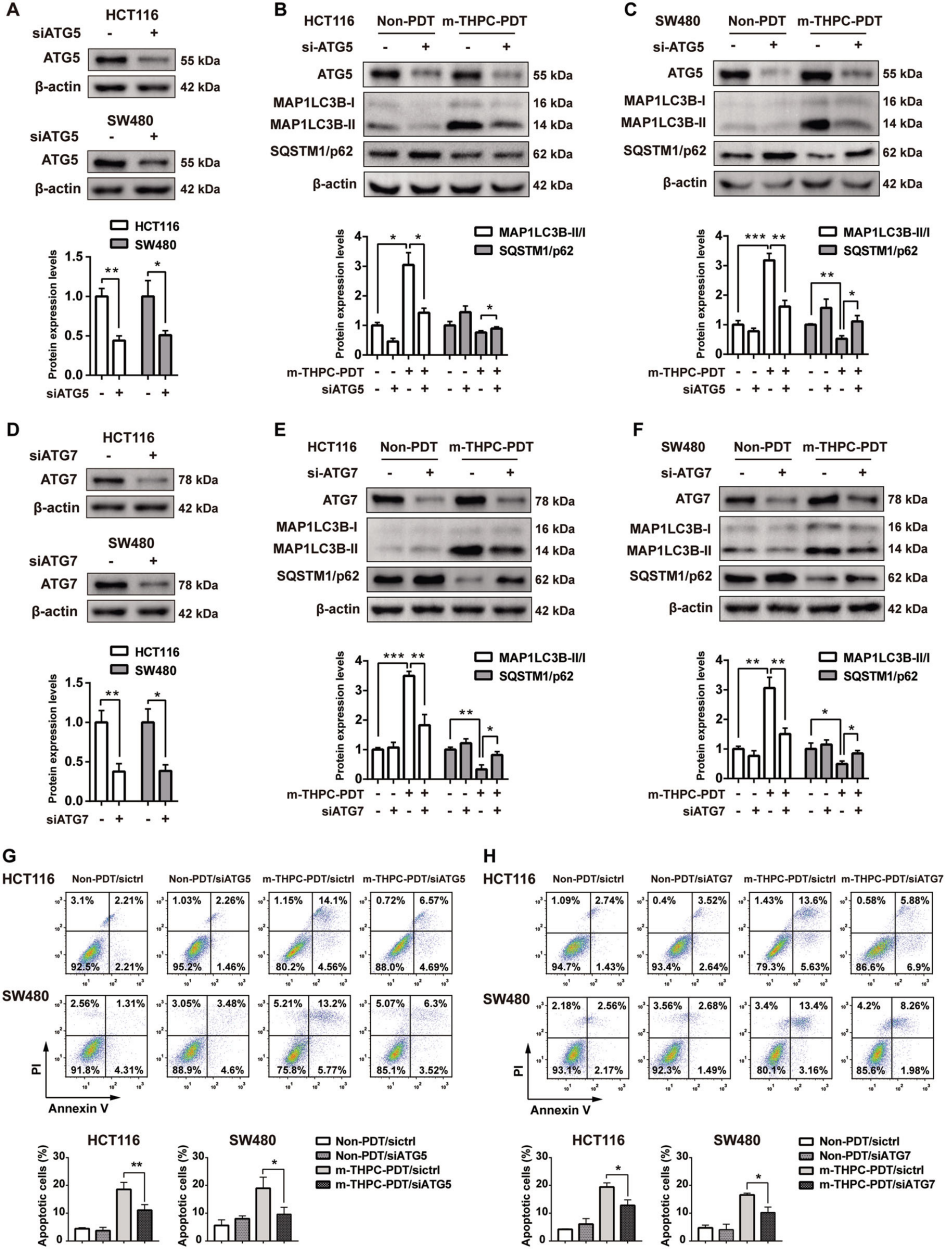
Knockdown of ATG5 or ATG7 relieved the apoptosis induced by m-THPC-PDT in CRC cells. HCT116 and SW480 cells were transfected with 50 nmol/L siATG5 or siATG7 for 48 h, respectively. After that, cells were treated with 0.7 μmol/L m-THPC for 8 h, and then irradiated with a light dose of 3 J/cm^2^, followed by incubation without irradiation for 16 h. **A**–**F** Western blot analysis of ATG5, ATG7, MAP1LC3B-I, MAP1LC3B-II, and SQSTM1/p62 levels in HCT116 and SW480 cells. **G**, **H** Flow cytometry was performed to measure cell apoptosis. All data are presented as the mean ± SD, *n* = 3. ^*^P < 0.05, ^**^P < 0.01.

It has been shown that ATG5 and ATG7, two major autophagy-related proteins, are important components in the formation of autophagosomes^38^. To determine the role of autophagy in m-THPC-PDT in CRC cells, we used siRNAs to downregulate ATG5 and ATG7 genes in CRC cells. As shown in Fig. 5A, D, siATG5 or siATG7 significantly decreased the level of ATG5 or ATG7 in both HCT116 and SW480 cells, respectively. In addition, downregulation of ATG5 or ATG7 inhibited the m-THPC-PDT-induced autophagy in CRC cells, as indicated by the m-THPC-PDT-induced increase in MAP1LC3B-II expression and a decrease in SQSTM1/p62 expression that could be counteracted by ATG5 or ATG7 downregulation (Fig. 5B, C, E, F). Moreover, knockdown of ATG5 or ATG7 effectively reversed the apoptosis in CRC cells induced by m-THPC-PDT (Fig. 5G, H). These data illustrated that inhibition of autophagy could abolish the apoptosis induced by m-THPC-PDT in CRC cells.

### M-THPC-PDT treatment inhibited tumorigenesis of HCT116 subcutaneous xenografts in vivo by inducing autophagy

**Fig. 6:**
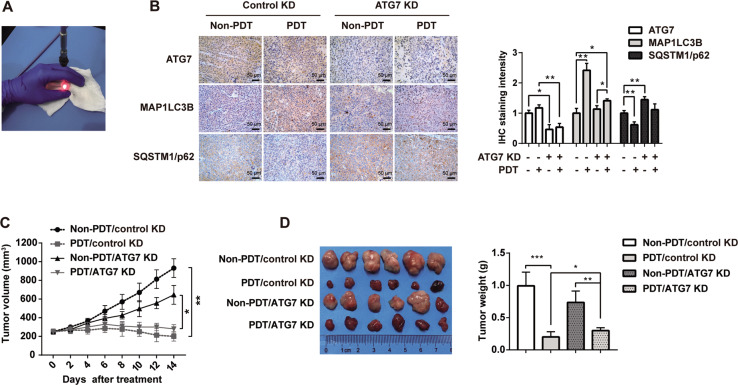
M-THPC-PDT treatment inhibited tumorigenesis of HCT116 subcutaneous xenografts in vivo by inducing autophagy. **A** Image of PDT therapy in CRC tumor-bearing nude mice. **B** Representative IHC images of ATG7, MAP1LC3B, and SQSTM1/p62 in tumor tissues (40× magnification). **C** The tumor volume of each animal was measured every other day. **D** Fourteen days post m-THPC-PDT treatment, xenograft tumors were photographed. All data are presented as the mean ± SD. ^*^P < 0.05, ^**^P < 0.01, *n* = 6 mice per group.

To investigate the in vivo therapeutic efficiency of m-THPC-PDT, HCT116 subcutaneous xenograft models were established. Figure 6A shows PDT therapy in CRC tumor-bearing nude mice. As indicated in Fig. 6C, D, m-THPC-PDT treatment significantly reduced the tumor volume of HCT116 subcutaneous xenografts compared with the non-PDT group. However, the inhibitory effect of m-THPC-PDT on tumor volume was relieved by the knockdown of ATG7 (Fig. 6C, D). Moreover, IHC assays revealed that m-THPC-PDT treatment obviously increased the expression of MAP1LC3B-II and decreased the expression of SQSTM1/p62 in tumor tissues, indicating that m-THPC-PDT could induce autophagy in a xenograft model. Conversely, downregulation of ATG7 reduced m-THPC-PDT-induced autophagy in tumor tissues, as revealed by the m-THPC-PDT-induced increase in MAP1LC3B-II protein expression and a decrease in SQSTM1/p62 expression that could be counteracted by ATG7 downregulation (Fig. 6B). These results indicated that inhibiting autophagy could relieve the anti-tumorigenic properties of m-THPC-PDT in a HCT116 subcutaneous xenograft model.

### PDT treatment triggered apoptosis and autophagy by activating the ROS/JNK signaling pathway

**Fig. 7:**
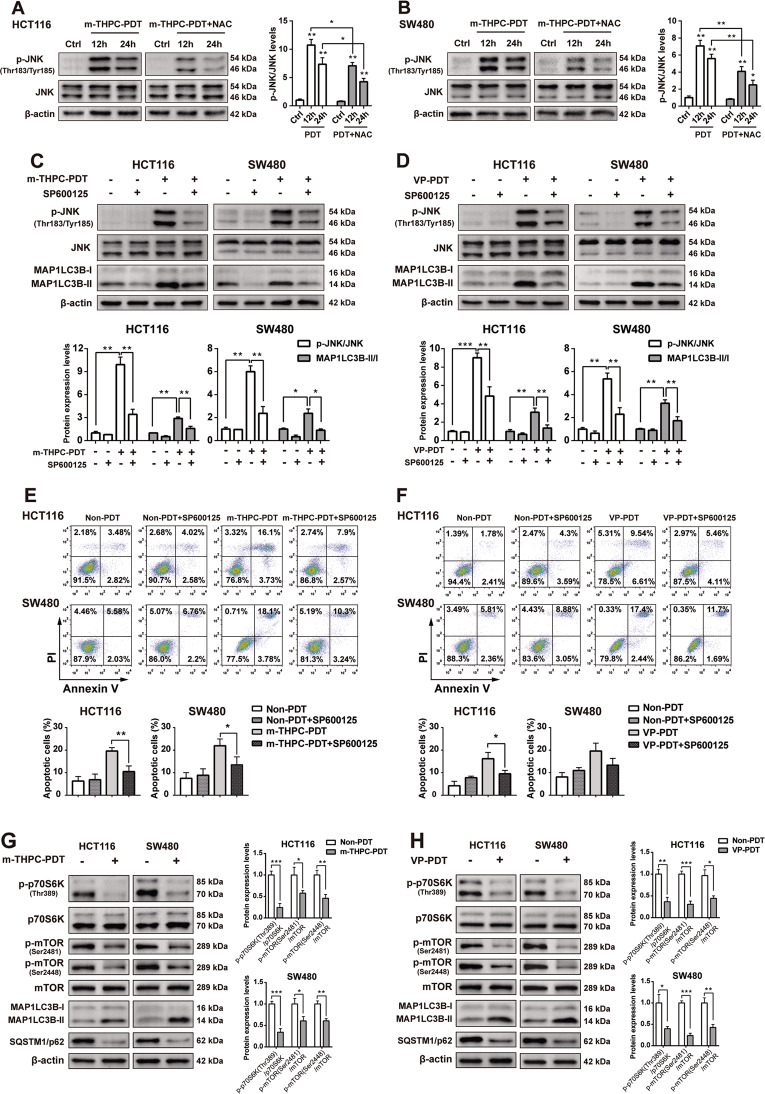
PDT treatment triggered autophagy by activating the ROS/JNK signaling pathway and inhibiting the mTOR signaling pathway. **A** HCT116 and **B** SW480 cells were treated with 0.7 μmol/L m-THPC and 5 mmol/L NAC for 8 h, and then irradiated with a light dose of 3 J/cm^2^, followed by incubation without irradiation for another 12 or 24 h. Western blot analysis of p-JNK (Thr183/Tyr185) and JNK levels in HCT116 and SW480 cells. HCT116 and SW480 cells were treated with **C** 0.7 μmol/L m-THPC or **D** 0.35 μmol/L VP in the presence or absence of 10 μmol/L SP600125 for 8 h, and then irradiated with a light dose of 3 J/cm^2^, followed by incubation without irradiation for 16 h. Western blot analysis of p-JNK, JNK, MAP1LC3B-I, and MAP1LC3B-II levels in HCT116 and SW480 cells. **E**, **F** Flow cytometry was applied to measure cell apoptosis. **G**, **H** HCT116 and SW480 cells were treated with 0.7 μmol/L m-THPC or 0.35 μmol/L VP, respectively, for 8 h, and then irradiated with a light dose of 3 J/cm^2^, followed by incubation without irradiation for 8 h. Western blot analysis of p-p70S6K (Thr389), p70S6K, p-mTOR (Ser2481), p-mTOR (Ser2448), mTOR, MAP1LC3B-I, MAP1LC3B-II, and SQSTM1/p62 levels in cells. All data are presented as the mean ± SD, *n* = 3. ^*^P < 0.05, ^**^P < 0.01.

To determine whether PDT-induced autophagy and apoptosis are related to ROS/JNK signaling, NAC (a ROS scavenger), and the JNK inhibitor SP600125 were used for further investigation. As indicated in Fig. 7A, B, m-THPC-PDT treatment remarkably increased the phosphorylation of JNK in CRC cells. Conversely, incubation with NAC effectively reduced the phosphorylation of JNK in m-THPC-PDT-treated CRC cells, indicating that ROS might be the upstream signal molecule of JNK in CRC cells treated with m-THPC-PDT (Fig. 7A, B). In addition, JNK inhibitor SP600125 prevented the phosphorylation of JNK and the formation of MAP1LC3B-II in PDT-treated CRC cells (Fig. 7C, D). Moreover, SP600125 markedly inhibited m-THPC-PDT- or VP-PDT-induced apoptosis in CRC cells (Fig. 7E, F). These data indicated that PDT treatment triggered apoptosis and autophagy in CRC cells by activating the ROS/JNK signaling pathway.

### PDT treatment induced autophagy by inhibiting the mTOR signaling pathway

**Fig. 8:**
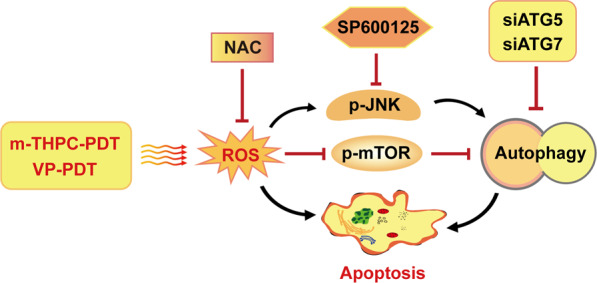
Diagram of the mechanism of PDT inducing autophagy and apoptosis in CRC cells. M-THPC-PDT or VP-PDT treatment results in ROS accumulation, which induced autophagy and apoptosis in CRC cells, through regulating JNK and mTOR pathways. Moreover, NAC, SP600125, or siATG5/siATG7 can inhibit autophagy, which reduces the apoptosis induced by PDT.

To determine whether PDT-induced autophagy is related to mTOR signaling, the protein expression of phospho-mTOR (p-mTOR) and phospho-p70S6K (p-p70S6K) were detected. As shown in Fig. 7G, H, m-THPC-PDT or VP-PDT treatment resulted in decreased phosphorylation of p70S6K and mTOR, elevated autophagic MAP1LC3B-II/MAP1LC3B-I ratio, and decreased levels of SQSTM1/p62 in CRC cells. These data suggested that PDT treatment induced autophagy by inhibiting the mTOR signaling pathway (Fig. 8).

## Discussion

As a minimally invasive technology used for the treatment of tumors, PDT involves a combination of a PS, molecular oxygen, and light to trigger cell death^39^. M-THPC and VP are two second-generation photosensitizers: m-THPC has been mainly used in the treatment of patients with head and neck cancer, while VP has been primarily used in the treatment of patients with age-related macular degeneration^40,41^. Recently, evidence has shown that m-THPC and VP exhibited excellent antitumor efficiency in CRC cells^16,42^. In this study, we found that m-THPC-PDT and VP-PDT had a strong phototoxic effect on CRC cells, as shown by decreased cell viability and increased ROS production. In addition, m-THPC-PDT treatment significantly inhibited the tumor growth of HCT116 xenografts in vivo. These data indicated that PDT treatment could suppress the growth of CRC cells in vitro and in vivo.

Evidence has shown that a PS is activated via light exposure to produce ROS and induce oxidative stress. PS-mediated PDT further induces cell apoptosis and autophagy in cancer cells^31,43,44^. In this study, flow cytometry assays indicated that m-THPC-PDT markedly induced the apoptosis of CRC cells. In addition, autophagy is an important cellular catabolic pathway that controls cell death^45^. Therefore, to investigate the role of PS-mediated PDT in autophagy, the markers of autophagy and autophagosomes were assessed by western blot and fluorescence microscopy. Our data showed that m-THPC-PDT and VP-PDT treatment induced autophagy in CRC cells, as shown by an increased ratio of MAP1LC3B-II/MAP1LC3B-I and the degradation of the SQSTM1/p62 protein. PDT treatment markedly induced the formation of GFP-MAP1LC3B-II puncta in CRC cells. Furthermore, three autophagy inhibitors, BafA1, CQ, and 3-MA, were used to confirm whether PDT treatment induced autophagy in CRC cells. We found that inhibition of autophagy abolished the autophagy induced by m-THPC-PDT or VP-PDT in CRC cells. These data suggested that m-THPC-PDT and VP-PDT treatment triggered apoptosis and autophagy in CRC cells.

The mechanisms regulating apoptosis and autophagy are complicated^43^. Depending on the ROS level, PS, and cell types, autophagy may be cytotoxic or cytoprotective^46^. Zhu et al.^31^ indicated that inhibition of autophagy enhanced sinoporphyrin sodium mediated-PDT (DVDMS-PDT) induced apoptosis in CRC cells, indicating that autophagy was protective in DVDMS-PDT-treated CRC cells. In addition, Lange et al. indicated that m-THPC-PDT could induce cell death and autophagy in human cancer cells, and found that autophagy might occur in parallel to apoptosis or that autophagy might be dominant, eventually resulting in autophagy-associated apoptosis^47^. Huang et al.^43^ revealed that inhibition of autophagy decreased the apoptosis induced by pyropheophorbide-α methyl ester-mediated PDT in osteosarcoma cells. These data indicate that autophagy can also exhibit a cytotoxic effect. In this study, to determine the role of m-THPC-PDT- and VP-PDT-induced autophagy in CRC cells, CRC cells were transiently transfected with either ATG5 siRNA or ATG7 siRNA. Our data showed that the inhibition of m-THPC-PDT-induced autophagy by knockdown of ATG5 or ATG7 inhibited the apoptosis of CRC cells. Moreover, the inhibitory role of m-THPC-PDT on tumor volume was relieved by the knockdown of ATG7 in vivo. Our data indicated that inhibition of autophagy could alleviate the PDT-mediated anticancer efficiency in CRC cells, and that autophagy may exhibit a potent cytotoxic effect on m-THPC-PDT- or VP-PDT-treated CRC cells.

Evidence has shown that the ROS-JNK signaling pathway participates in PDT-induced autophagy and apoptosis^43^. ROS can promote autophagy by activating JNK signaling^48^. To determine whether PDT-induced autophagy and apoptosis are related to ROS/JNK signaling, NAC (a ROS scavenger) and the JNK inhibitor SP600125 were used. Our data indicated that m-THPC-PDT significantly increased the phosphorylation of JNK in CRC cells, which was reversed in the presence of NAC. These data indicated that JNK acted as one of the targets of ROS. In addition, SP600125 significantly inhibited autophagy and apoptosis induced by m-THPC-PDT in CRC cells. These data indicated that PDT treatment triggered apoptosis and autophagy in CRC cells by activating the ROS/JNK signaling pathway.

The mTOR/p70S6K signaling pathway plays an important role in the development of human cancer, and it can negatively regulate autophagy^49^. Inhibition of mTOR signaling could induce autophagy and apoptosis^50,51^. In this study, we found that m-THPC-PDT or VP-PDT treatment caused a decreased phosphorylation of p70S6K and mTOR in CRC cells. These data suggested that PDT treatment could induce autophagy by inhibiting of the mTOR signaling pathway.

## Conclusion

Our study demonstrated that PDT treatment induces apoptosis and autophagy in CRC cells by upregulating ROS, activating JNK pathway and inhibiting mTOR/p70S6K pathway. In addition, inhibition of autophagy can remarkably abolish PDT-mediated anticancer efficiency in CRC cells. Altogether, m-THPC and VP might be promising PSs, and our study provides evidence for the therapeutic application of m-THPC and VP in CRC.
